# Delivery mode and maternal gestational diabetes are important factors in shaping the neonatal initial gut microbiota

**DOI:** 10.3389/fcimb.2024.1397675

**Published:** 2024-08-29

**Authors:** Xuan Shi, Yanfang Liu, Teng Ma, Hao Jin, Feiyan Zhao, Zhihong Sun

**Affiliations:** ^1^ Inner Mongolia Key Laboratory of Dairy Biotechnology and Engineering, Inner Mongolia Agricultural University, Hohhot, Inner Mongolia, China; ^2^ Key Laboratory of Dairy Processing, Ministry of Agriculture and Rural Affairs, Inner Mongolia Agricultural University, Hohhot, Inner Mongolia, China; ^3^ Key Laboratory of Dairy Biotechnology and Engineering, Ministry of Education, Inner Mongolia Agricultural University, Hohhot, Inner Mongolia, China; ^4^ Collaborative Innovation Center of Lactic Acid Bacteria and Fermented Dairy Products, Ministry of Education, Inner Mongolia Agricultural University, Hohhot, Inner Mongolia, China

**Keywords:** gut microbiota, infant, maternal and neonatal, bacteriophage, delivery method

## Abstract

**Background:**

The infant gut microbiome’s establishment is pivotal for health and immune development. Understanding it unveils insights into growth, development, and maternal microbial interactions. Research often emphasizes gut bacteria, neglecting the phageome.

**Methods:**

To investigate the influence of geographic or maternal factors (mode of delivery, mode of breastfeeding, gestational diabetes mellitus) on the gut microbiota and phages of newborns, we collected fecal samples from 34 pairs of mothers and their infants within 24 hours of delivery from three regions (9 pairs from Enshi, 7 pairs from Hohhot, and 18 pairs from Hulunbuir) using sterile containers. Gut microbiota analysis by Shotgun sequencing was subsequently performed.

**Results:**

Our results showed that geographic location affects maternal gut microbiology (*P* < 0.05), while the effect on infant gut microbiology was not significant (*P* = 0.184). Among the maternal factors, mode of delivery had a significant (*P* < 0.05) effect on the newborn. Specific bacteria (e.g., *Bacteroides*, *Escherichia* spp., *Phocaeicola vulgatus*, *Escherichia coli*, *Staphylococcus hominis*, *Veillonella* spp.), predicted active metabolites, and bacteriophage vOTUs varied with delivery mode. *Phocaeicola vulgatus* significantly correlated with some metabolites and bacteriophages in the early infant gut (*P* < 0.05). In the GD group, a strong negative correlation of phage diversity between mother and infants was observed (*R* = -0.58, *P*=0.04).

**Conclusion:**

In conclusion, neonatal early gut microbiome (including bacteria and bacteriophages) colonization is profoundly affected by the mode of delivery, and maternal gestational diabetes mellitus. The key bacteria may interact with bacteriophages to influence the levels of specific metabolites. Our study provides new evidence for the study of the infant microbiome, fills a gap in the analysis of the infant gut microbiota regarding the virome, and emphasizes the importance of maternal health for the infant initial gut virome.

## Introduction

Microorganisms, the earliest life forms on Earth, inhabit the entire world where humans reside. Among the human body’s microbiota, the gastrogut tract stands as the most densely populated, harboring an estimated count of over 10^14^ microorganisms (Thursby and Juge, 2017). Predominantly, bacteria dominate this environment, alongside fungi, viruses, archaea, and protozoa. Among these, bacteria and viruses (primarily bacteriophages) have been extensively researched (Shkoporov et al., 2022). It has become evident that the structure and composition of diverse gut microorganisms significantly influence a plethora of vital physiological activities, including nutrient processing, metabolism, energy balance, and the development of the host’s immune system (Choi et al., 2021).

The existence of microbiome colonization in humans before birth is a controversial topic. For more than a century, the scientific community has considered the uterus to be “sterile” (Willyard, 2018). Until 2014, Professor Aagaard found that the placenta contained small amounts of bacteria similar to the oral microbiota, speculating that neonatal microbiota colonization may begin before birth (Agans et al., 2011). In recent years, several studies have used microbiological methods such as polymerase chain reaction amplification and bacterial culture to investigate the human placenta and have not found any evidence to support the presence of a placental microbiota (Theis et al., 2019; Kennedy et al., 2021; Sterpu et al., 2021), but the possible presence of viruses (De Goffau et al., 2019). The bacterial signal detected in some amniotic fluid and placenta (Mishra et al., 2021) may come from different contaminations, such as sampling equipment, personnel and environment, etc (Kennedy et al., 2023). However, it is nevertheless undeniable that from the moment of birth, the gut microbiota immediately becomes rapidly colonized by microorganisms from the maternal and surrounding environment (Laursen et al., 2021), and that the survival of colonized gut microorganisms early in life is associated with the potential development of disease in childhood and even in adulthood (Ronan et al., 2021), such as food allergies, asthma, atopic eczema, metabolic disorders, and inflammatory bowel disease. The opinion that “neonatal gut microbiota is maternal in origin” has been widely accepted as studies continue to be published. A recent study found that 58.5% of infant microbiota originated from maternal vaginal, fecal, skin, breast milk, nasopharyngeal, and saliva microbiota (with breast milk microbiota making the majority contribution), and that all maternal sites provided microbiota ecological niches for multiple sites of infant microbiota (Bogaert et al., 2023). The transmission of the maternal microbiota to the infant’s gut microbiota is influenced by various factors, including the mode of delivery, mode of feeding, maternal health, and antibiotic usage (Wang et al., 2020a). Of these factors, both the mode of delivery and mode of feeding play significant roles in shaping the infant’s gut microbiota (Mitchell et al., 2020; Pärnänen et al., 2022). For example, infants delivered via caesarean section often exhibit lower levels of *Bacteroides*, *Bifidobacterium*, and *Parabacteroides* species in their gut tract. Conversely, there is a comparatively higher presence of opportunistic pathogens, which are frequently associated with the hospital environment (Xiao and Zhao, 2023). In infants delivered via cesarean section, there can be an interruption in the transmission of *Bacteroidetes*, which consequently leads to a delay in the colonization of the gut by pioneer bacteria, when compared to infants born through vaginal deliveries (Shao et al., 2019). Of greater importance, the species inherited by infants from their mothers have a direct association with the early development of crucial gut functions, including the capability to metabolize and utilize breast milk oligosaccharides (Ma et al., 2022). These inherited strains predominantly originate from the transfer of the mother’s gut microbiota (Wang et al., 2020b). Although research has shown that the impact of the microbiota gap caused by the mode of delivery diminishes over time or can be mitigated by breastfeeding, its differences remain apparent even at the 8-month mark (Shao et al., 2019). This indicates that the mode of delivery has a substantial influence on the development of the neonatal gut.

The early development of the gut microbiota during the first years of life plays a pivotal role in the maturation of the infant’s immune system (Backhed et al., 2015). Based on most reported studies, the neonatal gut microbiome primarily consists of obligate anaerobic bacteria and facultative anaerobic bacteria, which play a leading role in shaping the composition of the infant gut microbiota. These pioneers mainly include *Enterococcus*, *Bacteroides*, *Lactobacillus*, *Staphylococcus* and *Enterobacter*, etc (Van de Vliet and Joossens, 2022). While existing studies have primarily concentrated on the bacterial constituents of the gut microbiome, it is essential to acknowledge that the virome is also a significant member of this microbial community. During the initial months of life, bacteriophages, similar to bacteria, exhibit a host-specific distribution within the gut and play a crucial role in regulating bacterial growth through mechanisms such as lysis and lysogeny (Shah et al., 2023). In 2008, the first study investigating the composition of viral metagenomic in the infant gut revealed that the viral community of a one-week-old infant displayed minimal diversity and was primarily dominated by bacteriophages. This dominance of bacteriophages could potentially impact the diversity and abundance of coexisting microorganisms (Breitbart et al., 2008). In a recent study involving a substantial cohort of fecal virus metagenomes from one-year-old children, researchers identified 10,000 viruses belonging to 248 virus family-level clades (VFCs). It is noteworthy that the majority of these VFCs were previously undiscovered and classified under the Caudoviricetes virus class (Shah et al., 2023). However, comprehensive investigations into the composition and structure of viral groups during early life remain scarce, and the study of human viral groups is typically challenged by the vast diversity of unknown viruses.

Therefore, we collected fecal samples from mothers and infants (within 24 hours after delivery) from three different regions in China, and performed metagenomic sequencing with a data volume of 20G per sample to investigate the effects of different factors on the diversity and structural composition of the gut microbiome and bacteriophage in newborns.

## Materials and methods

### Ethics approval

This study was approved by the Special Committee on Scientific Research and Academic Ethics of Inner Mongolia Agricultural University (No. 2018-059). The present investigation was in accordance with the principles outlined in the Declaration of Helsinki. Informed consent was obtained from all recruited subjects prior to the start of the study. For confidentiality and data protection of participant information, we protect participants’ identities by replacing participant identifiers with codes and storing the data electronically on password-protected computers, and by deleting or hiding personal information from the data when it is uploaded.

### Experimental design and cohort recruitment

Our previous research has shown significant differences in the composition of gut microbiota between urban and pastoral populations in Inner Mongolia (Zhang et al., 2013). Additionally, Sequencing of fecal samples from 2,678 healthy Chinese people from 28 provinces in China revealed that geography explains the largest microbiota variations (Lu et al., 2021). Therefore, from July 2018 to May 2019, we recruited a total of 34 Chinese pregnant women (7 in Hohhot, 9 in Enshi, and 18 in Hulunbuir) in Enshi Yafia Maternity Hospital (Enshi, Hubei Province), Inner Mongolia Autonomous Region People’s Hospital (Hohhot, Inner Mongolia) and Hulunbuir Maternal and Child Health Hospital (Hulunbuir, Inner Mongolia). Both Enshi and Hohhot are urban areas, while Hulunbuir is nomadic and more countryside oriented. Participants were requested to provide relevant information such as ethnicity, number of births, presence of gestational diabetes, as well as record the mode of delivery and breastfeeding status of each mother during the sampling period. Out of the 34 pregnant women included in the study, 10 opted for a vaginal delivery while 24 chose to undergo a cesarean section. Following delivery, 10 women opted for exclusive breastfeeding, while the remaining 24 women selected a mixed feeding (breastfeeding and formula feeding). 10 mothers had gestational diabetes mellitus, while the remaining 24 did not have gestational diabetes mellitus. Details of basic maternal information are shown in **Supplementary Table 1**.

### Sample collection

All samples were collected in sterile containers having an equal volume of sterile cryoprotectant. Hospital staff collected fecal samples from mothers within 24 hours of delivery using specific collection tubes for fecal material (Furui Biotechnology Co., Ltd., Guangzhou, China). All infant fecal samples were collected by the mothers themselves following detailed procedures explained by the doctor, and under the doctor’s supervision. Samples collected directly at the hospital were frozen at -20°C immediately after collection and transferred to a -80°C facility within one week, where they were stored until further analysis.

### DNA extraction and shotgun metagenomic sequencing

Shotgun sequencing was performed on 34 pairs of maternal feces and meconium samples from their infants collected within 24 hours of delivery. Metagenomic DNA was isolated from stool samples of patients utilizing the QIAamp Fast DNA Stool Mini Kit (Qiagen, Hilden, Germany). The DNA’s purity was evaluated by employing 1% agarose gel electrophoresis and a Nanodrop spectrophotometer, specifically measuring the 260 nm/280 nm ratio. Libraries were constructed using the NEBNext^®^ Ultra™ DNA Library Prep Kit for Illumina^®^ (E7370S/L; New England Biolabs, Ipswich, MA, USA) to generate DNA fragments of 300 bp, and paired-end reads were generated by sequencing 150 bp (Illumina HiSeq 2500; Illumina Inc., San Diego, CA, USA) in both forward and reverse directions. A total of 68 stool samples collected from 34 mother-infant pairs in three cities were metagenomically sequenced (Enshi: 18; Hohhot: 14; Hulunbuir: 36), generating 1.51 Tbp of high-quality paired-end reads (22.67 ± 1.81 Gbp per sample; range = 9.38-31.23 Gbp; **Supplementary Table 2**) for gut microbiota analysis.

### Reads assembly, contig binning, genome dereplication

The reads from each sample were assembled into contigs using MEGAHIT (Li et al., 2015). Contigs with a size greater than 2000 bp were selected for binning, employing MetaBAT2 (Kang et al., 2019), VAMB (Vollmers and Kindervater, 2010), SemiBin (Pan et al., 2022), MetaDecoder (Liu et al., 2022), and DAS Tool (Sieber et al., 2018) with default parameters. To obtain metagenome-assembled genomes (MAGs), the results generated by the five binners were combined using in-house scripts. Subsequently, the reads were mapped back to their respective contigs using BWA-MEM2 (Vasimuddin et al., 2019), and the contig depth was calculated using Samtools along with the jgi_summarize_bam_contig_depths function in MetaBAT2 (Li et al., 2009). The quality assessment of MAGs was performed using CheckM (Parks et al., 2015), which evaluated their completeness and contamination. Based on the criteria of completeness and contamination, MAGs were categorized into three groups: high-quality (completeness ≥80%, contamination ≤5%), medium-quality (completeness ≥70%, contamination ≤10%), and partial-quality (completeness ≥50%, contamination ≤5%). Subsequently, the high-quality MAGs were clustered, and dRep was employed to select the most representative genomes from each replicate set to extract species-level genome bins (SGBs) (Olm et al., 2017). This process was carried out with the parameters -pa 0.95 and -sa 0.95.

### Taxonomic annotation and SGB abundance analysis

The SGBs were subjected to annotation using Kraken2 and the NCBI NonRedundant Protein Sequence Database. The predicted genes were then compared against the UniProt Knowledgebase (UniProtKB, released in November 2020) using the blastp function of DIAMON, utilizing default options. To determine the relative abundance of each SGB, coverM (https://github.com/wwood/CoverM) was employed, with the parameter “-min-read-percent-identity 0.95 -min-covered-fraction 0.4”.

### Prediction of gut bioactive metabolites

The classification distribution of gut bioactive metabolite profiles in high-quality sequences was determined using the MelonnPan prediction process. Specifically, 1 million reads per sample were randomly selected using seqtk (https://github.com/lh3/seqtk) for subsampling. The subsampled reads were then compared using the blastx function of DIAMON with the parameters “-query-cover 90-id 50”. The best hit for each gene was chosen to calculate the gene abundance profile for each sample. Subsequently, the MelonnPan-predict workflow was applied to convert the gene abundances into a predicted metabolomic profiles.

### Bacteriophage contig identification, taxonomic annotation, and relative abundance analysis

After assembly by MEGAHIT, contigs larger than 1000bp were subjected to further viral characterization using VIBRANT and Checkv. By combining the results from both methods using an in-house script, a total of 54,604 potential viral sequences were identified. To filter the dataset, CD-HIT (https://github.com/weizhongli/cdhit) was employed to identify contigs larger than 5000bp with more than 95% nucleotide homology and over 80% coverage. Subsequently, 11,655 viral Operational Taxonomic Units (vOTUs) were obtained. These vOTUs were then compared to the Metagenomic Gut Virus catalogue (2021), and the average relative abundance was calculated using CoverM-contig pipeline (https://github.com/wwood/CoverM) with the following parameters: –min-read-percent-identity 0.95, –min-read-aligned-percent 0.5, –proper-pairs-only, and –exclude-supplementary.

### Statistical analyses

All statistical analyses were done using R software (v.4.2.0). To assess the microbiological outcomes, including species diversity, Principal Coordinates Analysis (PCoA), various R packages such as vegan, optparse, mixOmics, ggplot2, and ggpubr were employed. The significance of the differences was determined using Anosim and Adonis tests, with *P* values calculated based on 999 permutations. Wilcoxon rank-sum tests were utilized to evaluate variations in the abundance of strains, bacteriophages, metabolites, and other variables between different groups. To account for multiple testing, *P* values were corrected using the Benjamini-Hochberg procedure, and a corrected *P* value of less than 0.05 was considered statistically significant. Pearson’s correlation coefficient was employed to analyze the correlation between delivery-related metabolites and SGBs, with the results visualized using the corrplot R package. Procrustes analysis in the vegan package was used to determine similarity between two multivariate axes (permutations = 999). All graphical representations were created using the R and Adobe Illustrator software environments.

## Results

### Metagenome assembly strategies and general characteristics of genomes

**Figure 1:**
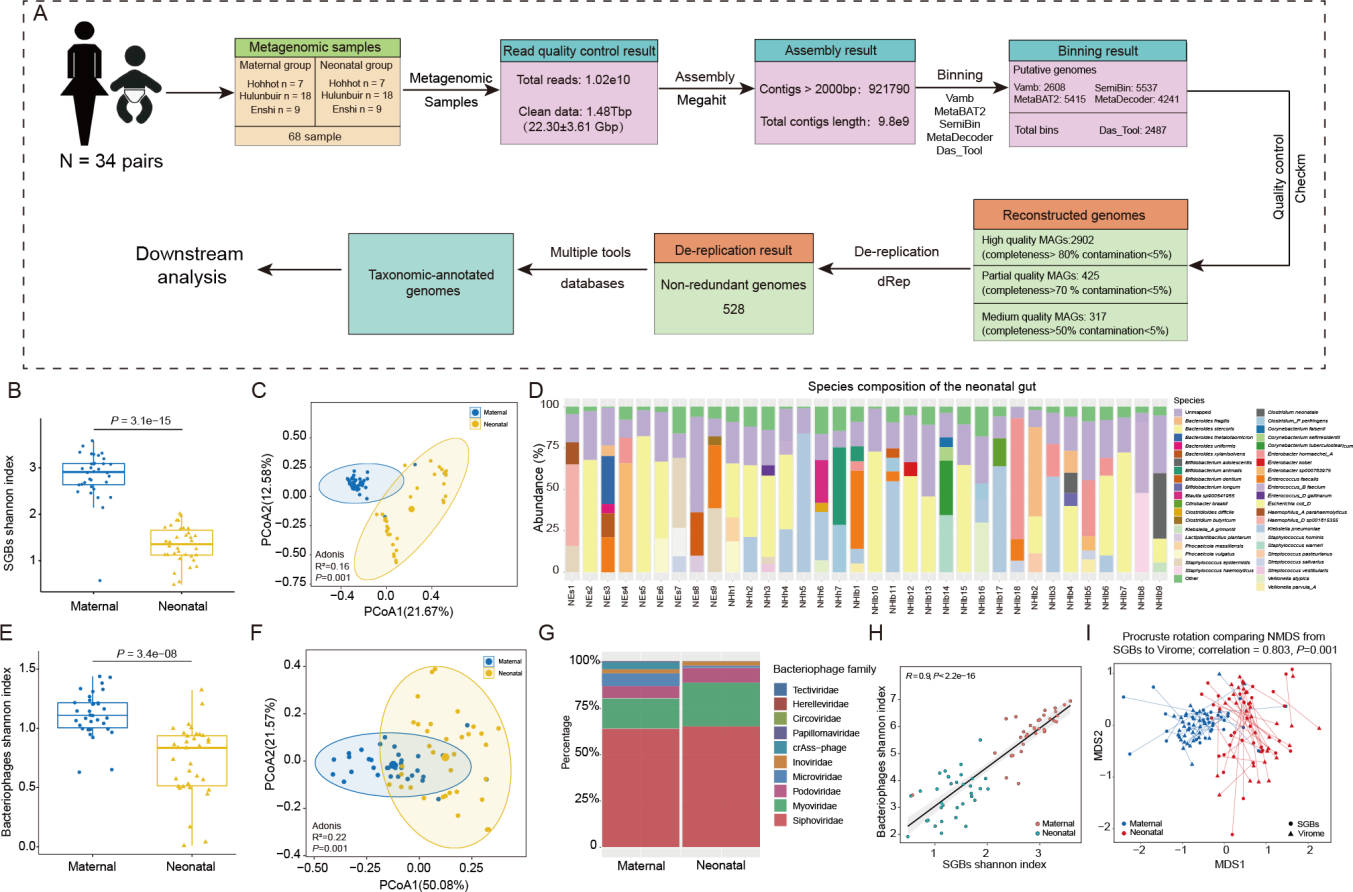
Metagenomic analysis pipeline and characterization of maternal - neonatal gut bacteria and bacteriophages. **(A)** The pipeline of metagenomic assembly, binning, reconstruction and dereplication. **(B)** Differences in the Shannon diversity index and **(C)** PCoA analysis of gut bacteria between mothers and infants. **(D)** Relative abundance of species-level genome bins (SGBs) in the gut of all infants, and the species with a proportion of 5% or less were added together and called Other. **(E)** Differences in the Shannon diversity index and **(F)** PCoA analysis of bacteriophages between mothers and infants. **(G)** Composition of maternal and neonatal gut bacteriophage in family-level taxonomic. **(H)** Correlation between the value of Shannon diversity index of the gut bacterial microbiota and virome; a strong positive correlation was found (*R* = 0.90; *P*< 0.001). **(I)** Procrustes analysis performed on gut species-level genome bins (SGBs) and virome of the mother and infant, showing a positive cooperativity between the gut bacterial and virome (correlation = 0.803; *P* = 0.001).

A total of 68 stool samples were subjected to sequencing, resulting in 1.51T of high-quality data (with an average of 22.67G per sample). The reads from each sample were assembled into contigs using megahits. Subsequently, macrogenomic binning was performed using MetaBAT2, VAMB, SemiBin, and MetaDecoder, yielding 5415, 2608, 5537, and 4241 bins, respectively. These original bins were then merged and refined using Das_Tool, resulting in 2487 bins. These bins were further classified using CheckM according to the latest standards, leading to the identification of 1646 high-quality genomes (**Supplementary Table 3**), 356 medium-quality genomes, and 390 local genomes. Finally, employing the principle of species-level genome nucleotide similarity, the high-quality genomes were compared and clustered. After selecting representative genomes from each cluster, a non-redundant set of 528 high-quality SGBs was obtained (**Figure 1A**; **Supplementary Table 4**). These 528 SGBs were distributed in 12 phyla, 18 orders, 43 orders, 84 classes, 272 genera, and 522 species. Comparison with the most recent database revealed that 491 of these SGBs had an Average nucleotide identity (ANI) of more than 95%, and 30 unnamed species were found in SGBs with ANI over 99% (n=86), which may be new species.

### Characterization and relationship between gut bacteria and bacteriophages in maternal/infant

Alpha diversity, principal coordinate analysis (PCoA) and Adonis analysis showed significant differences in gut bacteria diversity and structure between maternal and infant (*P* < 0.001, **Figures 1B, C**). The gut microbiota composition in the mother primarily consisted of Firmicutes (26.69%) and Bacteroidota (24.45%), whereas in the infant gut, Proteobacteria (44.44%) and Firmicutes (15.76%) were the dominant phyla. Moreover, at the genus level, the most prevalent genera in mothers included *Phocaeicola*, *Bacteroides*, *Escherichia*, and *Faecalibacterium*, among others. Whereas, infants were dominated by *Escherichia*, *Enterobacter*, *Staphylococcus*, *Bacteroides*, *Enterococcus*, and *Bifidobacterium* (**Supplementary Table 5**). Further analysis was conducted at the species level to provide more detailed insights. The results revealed that in the mother’s intestine, the dominant species included *Phocaeicola vulgatus*, *Escherichia coli_*D, *Bacteroides uniformis*, *Bacteroides stercoris*, *Prevotella copri*, and *Mediterraneibacter*. On the other hand, the infant gut was predominantly occupied by species such as *Escherichia coli*_D, *Klebsiella pneumoniae*, *Enterobacter hormaechei*_A, *Enterococcus faecalis*, *Staphylococcus epidermidis*, *Enterobacter* sp000762975, *Bacteroides fragilis*, and *Bifidobacterium animalis* (**Figure 1D**; **Supplementary Table 5**).

The constitution of gut microbiota is not only composed of bacteria, but bacteriophages also make up a significant portion of the population. Bacteriophages play an important role in late infancy cannot be understated, as this developmental stage marks a crucial period for the establishment and maturation of a child’s immune system (Shah et al., 2023). By interacting with bacteria, bacteriophages can have an impact on host health status (Barr, 2017). Therefore, we conducted an analysis of the diversity and composition of bacteriophages between mother and infant. Our bacteriophage search on the complete dataset returned a total of 11,654 non-redundant vOTUs. these vOTUs were then annotated with the metagenomic enterovirus catalog, assigning 65.39% of them to known bacteriophage families, including 147 complete genomes (1.92%), 71 high quality genomes (0.61%), and 1345 medium-quality genomes (17.65%) (**Supplementary Table 6**). A comprehensive analysis of the bacteriophage population revealed a total of 7,621 segments spanning 11 viral families, including Siphoviridae, Myoviridae, Microviridae, Podoviridae, and crAss-phage (**Supplementary Figure 1A**). The top 4 family levels being Siphoviridae (63.73%), Myoviridae (16.25%), Microviridae (6.91%), and Podoviridae (6.59%) for mothers and Siphoviridae (64.93%), Myoviridae (23.48%), Podoviridae (8.03%), and Inoviridae (2.38%) for infants, respectively (**Figure 1H**). Similar to the results of diversity analyses of bacteria, alpha-diversity and beta-diversity analyzes revealed significant differences in the bacteriophage structures of mothers and infants (**Figures 1F, G**).

Due to the predator-prey relationship between bacteriophages and bacteria, their interactions are also increasingly recognized as a factor influencing human gut ecology. The colonization and interactions between bacteriophages and bacteria early in life are of significance for future human health, but few studies have addressed them yet. We analyzed the phage and bacterial diversity of all samples for correlation, and found that showed a positive correlation in both mothers and infants (**Figure 1H**) This was confirmed by Procrustes correlation analysis of bacterial composition and phage composition (**Figure 1I**).

The human gut microbiota is established after birth. It is a dynamic process and stabilizes within 2-3 years. Affected by diet and aging, the complexity of the adult gut microbiota reaches the highest level. In addition, there are differences in the gut bacteriophages of infants and adults. At present, the question of when the infant intestine obtains the first batch of colonizers is still controversial. In addition, what microorganisms the infant can obtain from the mother’s intestine also needs more in-depth discussion. Although we did not find the vertical transmission of species in the mother-infant intestine, the analysis of bacteria-phage correlation showed positive synergy, which shows that bacteriophages are also one of the factors affecting the initial gut microbiota of infants. We will further explore the extent of the influence of geographical factors and maternal factors on infant gut microorganisms in the future.

### Geography has an effect over maternal gut microbiota and bacteriophage, but not infants

**Figure 2:**
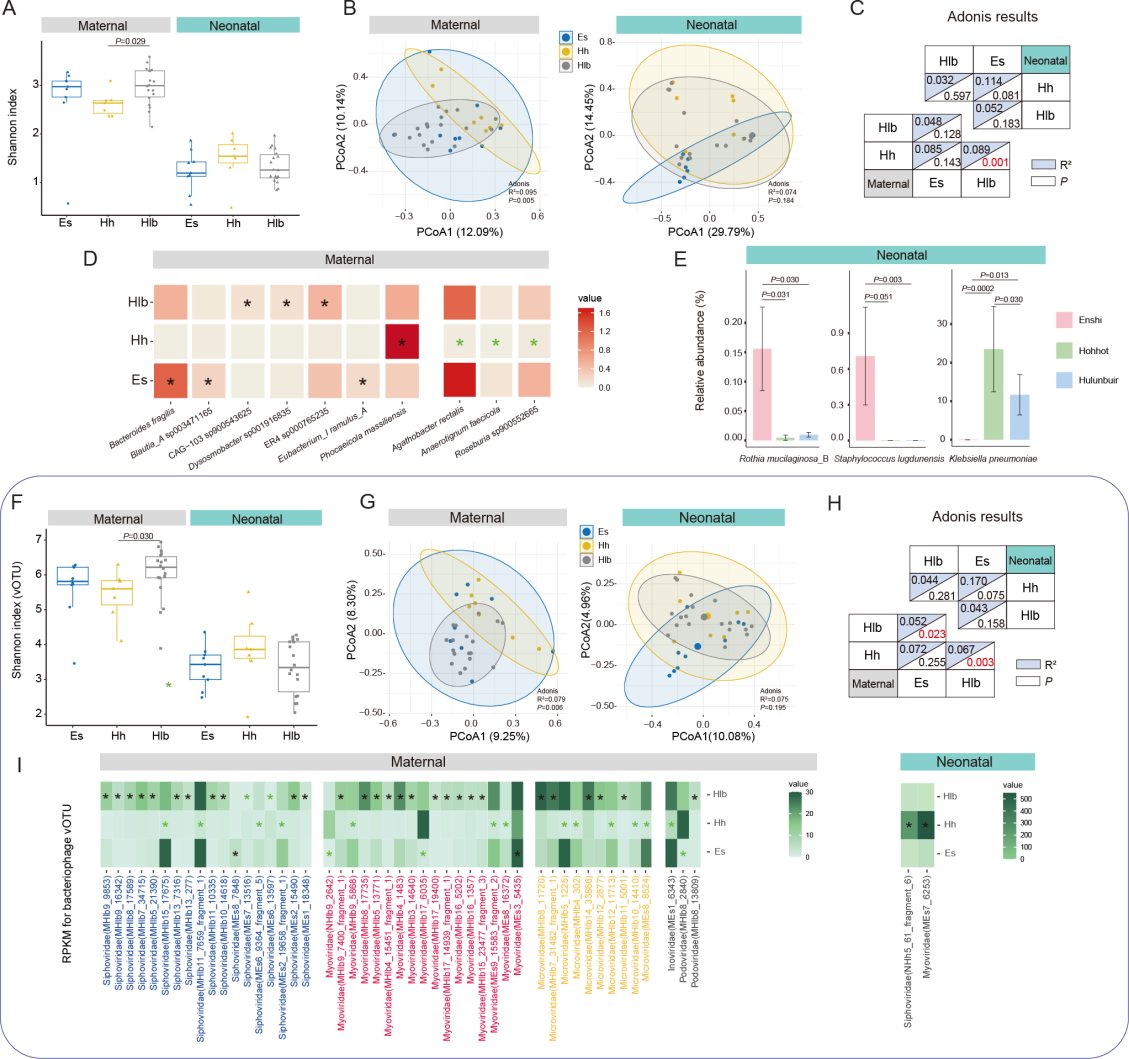
Characterization and differences in mothers/infant gut microbiomes between cities. **(A)** Shannon diversity index of mothers/infant gut bacteria in various cities. **(B)** PCoA analysis of gut bacteria in mothers/infants in different cities and **(C)** Adonis results between two-by-two cities. **(D)** Significant differential SGBs between mothers in various cities, with black “*” representing this city significantly higher than the other two and green “*” representing significantly lower than the other two. **(E)** Significant differential SGBs between infants in various cities. **(F)** Shannon diversity index of maternal/infant gut bacteriophage vOTUs in various cities. **(G)** PCoA analysis of gut bacteriophage vOTUs in mothers/infants in different cities and **(H)** Adonis results between two-by-two cities. **(I)** Significant differential gut bacteriophage vOTUs of mothers/infants in various cities. Es, Enshi; Hlb, Hulunbuir; Hh, Hohhot. RPKM, Reads Per Kilobase per Million mapped reads. Wilcoxon test was used to evaluate statistical differences; *P* < 0.05 was considered statistically significant [*P < 0.05; **P < 0.01].

Subsequently, we investigated the effect of geography on the gut microbiota of mothers or infants, respectively. Analysis performed by Shannon index showed significant differences in the alpha diversity of maternal gut bacteria between Hohhot and Hulunbuir (*P* = 0.029), while no significant differences were observed between the other cities (**Figure 2A**). The PCoA analysis demonstrated that the geography had a notable impact on the structure of the maternal microbiota (**Figure 2B**). Further pairwise analysis revealed that this significance primarily stemmed from the distinction between mothers in Hohhot and Hulunbuir (*P* = 0.001, **Figure 2C**). Regarding infants, no significant differences in Shannon’s index were found across all groups (**Figure 2A**), and no significant differences were observed in the structure of the infants’ microbiota across the three cities (**Figure 2C**). These findings suggest that geographic location exerts a certain influence on the diversity and structure of the gut microbiota in adults, while its impact on infants appears to be negligible. And this may be since newborns carry fewer microorganisms with a simpler bacterial community structure compared to adults, and thus are less affected by the geography and more affected by maternal factors as well as the hospital environment. This is consistent with our previous results that meconium samples exhibited lower within-group Bray-Curtis differences compared to maternal samples (He et al., 2020).

To explore species-level specific changes in the gut microbiota, we conducted a more detailed examination of differential species between mothers and infants from different regions. Among the mothers in the three regions, there were notable variations in the relative abundance of specific species in their gut microbiota. Mothers from Enshi exhibited significantly higher relative abundances of *Bacteroides fragilis*, *Blautia*_A sp003471165, and *Eubacterium_I ramulus*_A compared to mothers from the other two cities. What’s more, mothers from Hulunbuir showed significantly higher relative abundances of *Dysosmobacter* sp001916835, and two species of *Oscillospiraceae* (CAG-103 sp900543625, ER4 sp000765235) in their gut compared to mothers from the other two cities. Furthermore, *Phocaeicola massiliensis* displayed significantly higher abundance in the gut of mothers from Hohhot compared to the other two cities. On the contrary, *Agathobacter rectalis*, *Roseburia* sp900552665, and *Anaerotignum faecicola* exhibited significantly lower than other two cities (**Figure 2D**). Among the infants from the three regions, there were only a few differentially abundant species in the gut microbiota. *Rothia mucilaginosa*_B and S*taphylococcus lugdunensis* were found to be significantly higher in abundance in infants from Enshi compared to the other two cities. However, it should be noted that the abundance of *Staphylococcus lugdunensis* was zero in infants from Hohhot and Hulunbuir. On the other hand, *Klebsiella pneumoniae* showed significantly higher abundance in infants from Hohhot compared to the other cities, while its abundance was zero in infants from Enshi (**Figure 2E**; **Supplementary Table 8**). Overall, the presence of *Staphylococcus lugdunensis* and *Klebsiella pneumoniae* is regionally specific and not universal in all infant gut. Geographic factors have a much greater influence on the bacterial species of the mother’s gut than on the infant.

Then, we investigated the maternal and infant gut bacteriophages across the three regions. First, at the family level, there was a significant difference in alpha diversity only for mothers in Enshi and Hulunbuir (*P* = 0.041), with no difference between groups for infants (**Supplementary Figure 1B**). Whereas PCoA and adonis analyses showed that geography did not have a significant effect on bacteriophage family structure in mothers, the same was observed for infants (**Supplementary Figure 1C**). Therefore, we further performed the same analysis at the vOTU level. This time the results were still the same for infants, but showed different results between mothers than at the family level. In particular, the Shannon index turned out to be significant difference between Hohhot and Hulunbuir (*P* = 0.029), while there was no difference between the other groups (**Figure 2F**). Geography also showed a significant effect on maternal bacteriophage vOTU levels (*P* = 0.006, **Figure 2G**), with pairwise comparisons revealing a significant difference between Hulunbuir and the other two cities (*P* < 0.05, **Figure 2H**). To delve deeper into the potential differences between mothers and infants across cities, we investigated the bacteriophages family level (**Supplementary Table S9**). It was found that crAss-phage and Papillomaviridae exhibited significant differences only between mothers in Hohhot and Hulunbuir (*P* = 0.034; *P* = 0.029). Additionally, Inoviridae showed a significant difference solely between infants in Enshi and Hohhot (*P* = 0.031). However, no other statistically significant differences were observed in this analysis. Further searching for differential bacteriophages at the level of vOTUs (prevalence >10% and able to annotate to family), a total of 66 vOTUs (most belong to the dominant families, Siphoviridae, Myoviridae and Microviridae) were found between mothers in different regions, while infants had only 2 vOTUs (**Figure 2I**).

These results suggest that geography has an influence on the diversity and structure of adult gut bacteria and bacteriophage, especially between urban (Enshi and Hohhot) and countryside (Hulunbuir). Geography typically implies differences in living conditions, economic conditions, and dietary habits. It has been demonstrated that these factors tend to influence the gut microbiota of adults to a greater extent, the effect of which is far-reaching and difficult to change in a short period of time. In contrast, the neonatal gut microbial composition is relatively homogenous, so the effect of geographic factors on the infant’s gut bacteria and bacteriophage is minimal.

### Impact of different maternal factors on infant gut microbiome

Based on the aforementioned findings, the influence of geographic location on the gut microbiota and bacteriophage of infants was determined to be non-significant. And many reports also suggest that maternal factors can be a variable in early infant development. Consequently, we proceeded to investigate the potential effects of delivery mode, feeding mode, and maternal gestational diabetes (GD) on the structure and composition of the infant gut microbiota and bacteriophage.

**Figure 3:**
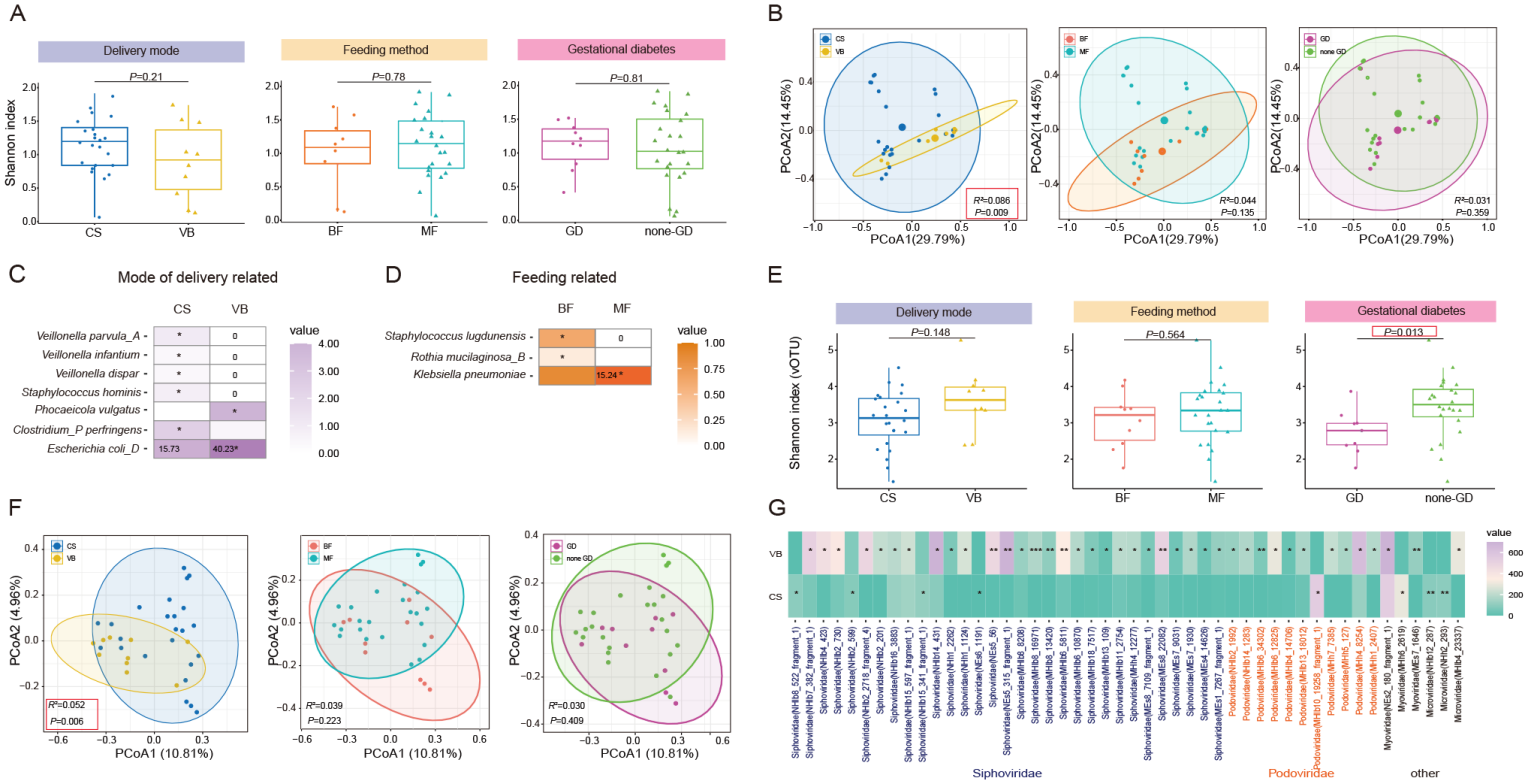
Characterization and differentiation of the infant gut microbiome under different maternal factors. **(A)** Shannon diversity index and **(B)** PCoA analysis of infant gut bacteria under different delivery mode, feeding mode, presence of Gestational diabetes in the mother. **(C)** Significant differential SGBs in the infant gut related to mode of delivery. **(D)** Significant differential SGBs in the infant gut related to mode of feeding. **(E)** Shannon diversity index and **(F)** PCoA analysis of infant gut bacteriophage vOTUs under different delivery mode, feeding mode, presence of Gestational diabetes in the mother. **(G)** Significant differential bacteriophage vOTUs in the infant gut related to mode of delivery. CS, Caesarean section; VB, Vaginal birth; BF, Breast feeding; MF, Mixed feeding; GD, Gestational diabetes; none-GD, None-Gestational diabetes. Wilcoxon test was used to evaluate statistical differences; *P* < 0.05 was considered statistically significant [*P < 0.05; **P < 0.01].

Alpha diversity analysis revealed that delivery mode, feeding mode, and maternal GD did not exert a notable impact on infant gut microbial diversity (**Figure 3A**). Beta diversity analysis demonstrated a significant difference in the gut microbial structure between infants delivered via vaginal birth (VB) and cesarean section (CS) (*P*=0.009). However, no significant differences were observed between breast feeding (BF) and mixed feeding (MF) groups, as well as between maternal GD and none-GD groups (**Figure 3B**). These findings indicate that among these factors, the mode of delivery has the most substantial influence on the structure of the infant gut bacteria, a conclusion further supported by the results of the anosim analysis (**Supplementary Figure 1E**).

Subsequently, we aimed to explore whether different factors could lead to changes in specific species within the gut microbiota. We identified 10 SGBs that exhibited significant differences between infants different delivery modes (**Figure 3C**; **Supplementary Table 8**). Among these, VB infants had significantly higher levels of *Clostridium_*AQ *innocuum*, *Bacteroides xylanisolvens*, *Bacteroides thetaiotaomicron*, *Phocaeicola vulgatus*, and *Escherichia coli*_D. It is noteworthy that *Escherichia coli* and *Bacteroides thetaiotaomicron* are considered opportunistic pathogens. *Clostridium*_P *perfringens*, *Veillonella parvula*_A, *Staphylococcus hominis*, *Veillonella infantium*, and *Veillonella dispar* had high levels in CS infants. In the comparison between exclusively BF and MF infants, we observed higher levels of *Corynebacterium kefirresidentii*, *Staphylococcus lugdunensis*, *Rothia mucilaginosa*_B, and *Streptococcus infantis*_I in the BF group. Conversely, *Klebsiella pneumoniae* exhibited higher levels in the MF group (**Figure 3D**; **Supplementary Table 8**). No significant differences in species were observed between infants in the maternal GD and maternal non-GD groups.

Compared to normal births, cesarean section infants have more exposure to their mother’s skin. Therefore, the initial microbiota they acquire is usually associated with skin microbiota such as *Veillonella*, *Staphylococcus*, *Corynebacterium*, *Propionibacterium* spp (Backhed et al., 2015). Similarly, breastfeeding infants are more likely to acquire these genera, especially Streptococcus and Staphylococcus, which are commonly found in the oral mucosa and skin surfaces. Large population analyses also confirmed that *Streptococcus* and *Staphylococcus*, as well as *Bifidobacterium*, are the pioneer bacteria in the gut habitat of most newborns in the early stages of life (Dwyer and Scharschmidt, 2022). In addition, the bacterial content of operating room dust was comparable to that of human skin bacteria (Shin et al., 2015). This suggests that skin microbes, as well as bacteria in the birthing environment, may be the first colonizers of infants. And the mode of delivery and feeding may affect the presence and abundance of these bacteria after delivery and throughout the early stages of life.

### Gestational diabetes affects the association between maternal-infant bacteriophages

Then, we conducted a further analysis to examine the impact of three factors on the diversity and composition of infant gut bacteriophages. The Shannon diversity index showed that infants in the none-GD group had higher gut bacteriophages diversity compared to those in the GD group (*P*=0.012). However, according to PCoA analysis, GD had no significant effect on the structure of bacteriophages in infants, and only the mode of delivery had a significant effect (*P*=0.006, **Figure 3F**). At the family level, we observed that only feeding patterns had a significant effect on bacteriophages levels. Specifically, BF infants displayed significantly higher levels of Podoviridae compared to MF infants (**Supplementary Table 9**). Dinleyici et al.’s analysis of the breast milk virome showed that Podoviridae were the most abundant family in transient human milk, with instead reduced abundance in mature breast milk. Given that our sampling time was about 24 hours after delivery, it is hypothesized that breastfed infants may acquire more Podoviridae in transient human milk (Dinleyici et al., 2021). At the vOTU level, we found more differences between groups (**Supplementary Table 10**). In particular, 48 differential vOTUs (31 to Siphoviridae, 11 to Podoviridae) were detected between delivery modalities, mostly (40/48) higher in the VB group (**Figure 3G**). There were 23 and 6 differential vOTUs found in the groups with different feeding methods and with or without GD, respectively (**Supplementary Figures 1F, S1G**).

Preceding results demonstrated that there is a positive bacteria-phages interaction (**Figure 1H**), and gestational diabetes had a significant effect on this relationship (*R* = 0.507, *P* = 0.003, **Supplementary Figure 1I**). Subsequently, we wanted to further explore whether the infant bacteriophages were associated with the mother’s bacteriophages/bacterial groups. We found that mother-infant pairs in the GD group were significantly negatively correlated with the Shannon index at the vOTU level (*R* = -0.65, *P* = 0.04, **Supplementary Figure 1J**), whereas there was no significant association in the none-GD group (*R* = 0.32, *P* = 0.13, **Supplementary Figure 1J**). This is consistent with the vOTU diversity analysis that GD mothers had higher vOTU diversity than none-GD mothers (*P* = 0.038, **Supplementary Figure 1D**), while the opposite was true for infants (*P* = 0.013, **Figure 3E**). In addition, we analyzed the Procrustes correlation between the mother’s and the infant’s bacteriophages composition. For mother-infant pairs in the GD or none-GD group, the bacteriophages composition between mothers and infants did not show a significant correlation at either the family level or the vOTU level. This implies that gestational diabetes may affect the diversity of mother and infant bacteriophages in different trends more than the structure of the phage.

In conclusion, there are different effects of different maternal factors on the diversity and structural composition of the infant’s gut microbiome, and this effect is similar in terms of species and phage vOTUs structure, again illustrating the positive correlation between the two in the host. Furthermore, we hypothesized that maternal gut virome influence the initial composition of the infant’s gut virome and are influenced by the health status of the mother. At a finer level of categorization, the effect of mode of delivery on the infant gut virome is more pronounced. We were unable to annotate these vOTUs due to technical limitations, but it can still be seen that the taxonomic level of the virus affects the results of the analysis. In subsequent studies, there is an urgent need to obtain finer phage classification information to make the analysis results clearer and more accurate.

### Effect of delivery mode on gut-predicted gut bioactive compounds in infants

**Figure 4:**
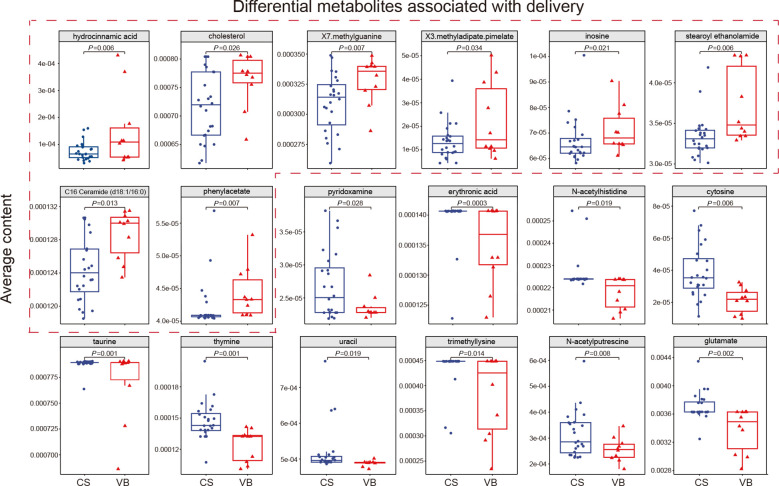
Differential metabolites associated with delivery. Average contents of 18 significantly different metabolites in CS infants and VB infants, with the 8 metabolites significantly higher in VB infants boxed in red dashed lines. CS, Caesarean section; VB, Vaginal birth. Wilcoxon test was used to evaluate statistical differences; *P* < 0.05 was considered statistically significant.

Among the four factors analyzed, including geography, delivery mode, feeding mode, and maternal gestational diabetes mellitus, only the delivery mode demonstrated a significant impact on the microbial composition and structure of the infant gut in this cohort. Consequently, we proceeded to explore the predicted biologically active metabolites in the guts of infants delivered via normal birth and cesarean section, aiming to discern any differences between the two delivery modes. The utilization of MelonnPan for prediction yielded 80 metabolites, with 18 of them showing differential levels associated with the mode of delivery. Among these metabolites, hydrocinnamic acid, cholesterol, X7. methylguanine, X3.methyladipate.pimelate, inosine, stearoyl ethanolamide, C16 Ceramide (d18:1/16:0), and Phenylacetate were found to have higher levels in VB infants. On the other hand, pyridoxamine, erythronic acid, N-acetylhistidine, cytosine, taurine, thymine, uracil, trimethyllysine, N-acetylputrescine, and glutamate displayed higher levels in CS infants (**Figure 4A**; **Supplementary Table 11**). The types of metabolites that were significantly higher in cesarean infants included pyrimidines, amino acids and their derivatives, pyridoxamine, polyamines and compounds of the glycolic acid group. Whereas, significantly higher in normal born infants include creatinine, purines, lipid molecules, organic compounds of aromatic carboxylic acids and amino acid metabolites. This suggested that differences in the mode of delivery may have an impact on the bacterial structure of the neonatal gut, which in turn affects distinct bacterial metabolite profiles.

### Key species and predicted gut bioactive compounds/bacteriophages interactions are influenced by mode of delivery

**Figure 5:**
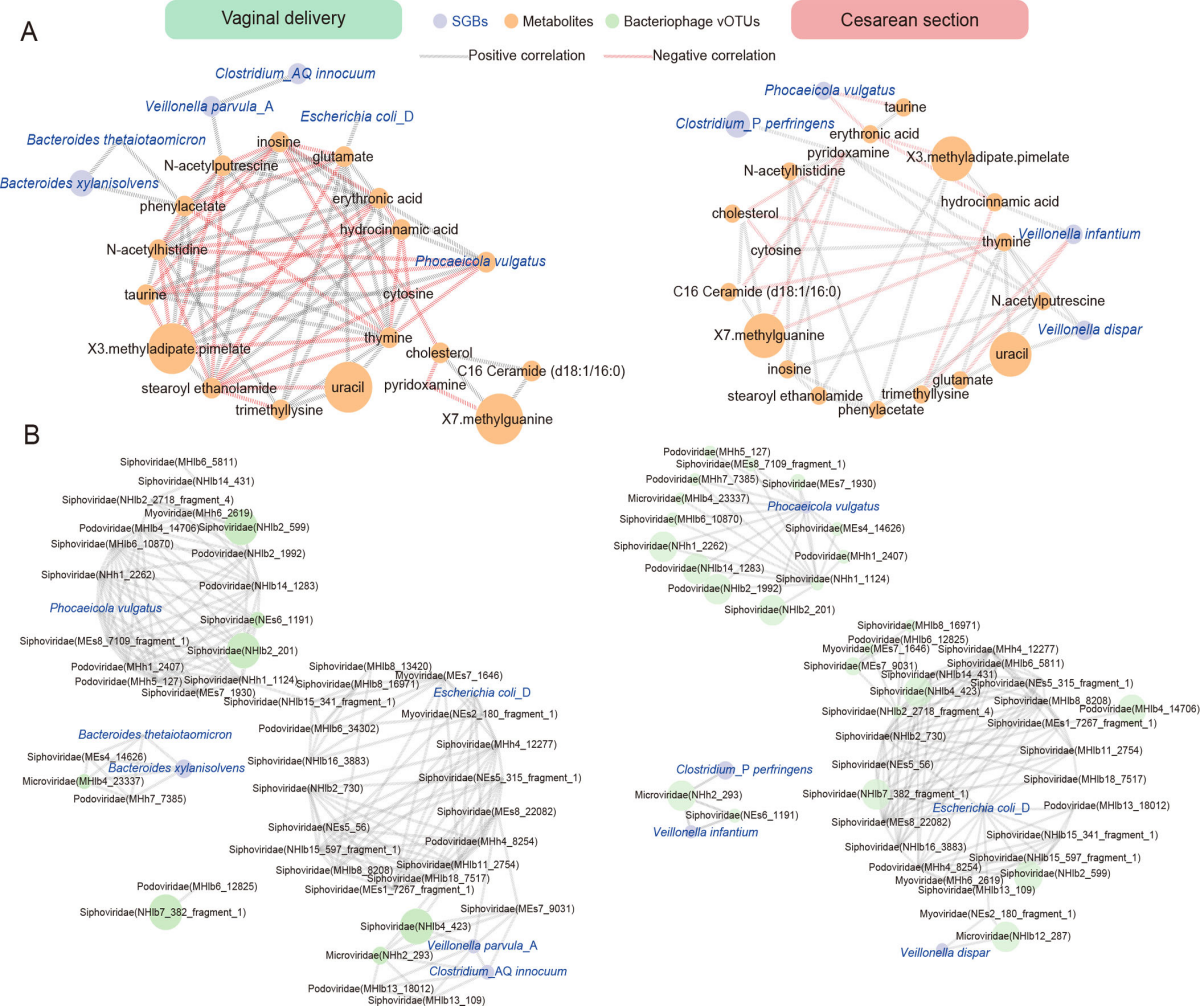
Correlation network analysis between differential SGBs and differential metabolites or bacteriophage associated with delivery. **(A)** Correlation analysis between 7 differential SGBs associated with delivery and 18 metabolites in CS infants and VB infants. **(B)** Correlation analysis between 7 differential SGBs associated with delivery and 48 bacteriophage vOTUs in CS infants and VB infants. The network was constructed based on the values of the Spearman rank correlation coefficients determined in the comparison of different SGBs and differential metabolites/bacteriophage. Edge width is proportional to correlation strength. The size of the nodes is proportional to the average abundance. The color of the line represents the correlation relationship, red - significant negative (*r* < − 0.2, *P* < 0.05), gray - significant positive (*r* > 0.2, *P* < 0.05).

We conducted a correlation analysis between the 18 differential metabolites associated with delivery modes and the 10 differential SGBs (**Supplementary Table 12**). We screened out the differential bacteria and metabolites with significant correlations (*P* < 0.05, |*r*| > 0.2) and plotted the correlation network. The figure demonstrated that the relationships between metabolite-metabolite, and bacteria-metabolite in the VB group are much more complex than in the CS group (**Figure 5A**). The findings revealed that in VB group, *Phocaeicola vulgatus* had the strongest correlations with metabolites. Specifically, there were significant positive correlations with hydrocinnamic acid, inosine, X3.methyladipate.pimelate (*r* > 0.2, *P* < 0.05), and significant negative correlations with cytosine, N-acetylhistidine, taurine, and thymine (*r* < -0.2, *P* < 0.05). Additionally, phenylacetate demonstrated a significant positive correlation with both *Bacteroides xylanisolvens* and *Bacteroides thetaiotaomicron*. On the other hand, in the CS group, *Veillonella infantium* and *Veillonella dispar* were significantly correlated with 3 metabolites, respectively (*P* < 0.05). In addition, *Phocaeicola vulgatus* showed significant negative correlation (*r* < -0.2, *P* < 0.05) with erythrosate and taurine. It was noteworthy that the presence of one species, *Phocaeicola vulgatus*, is associated with different metabolites in both groups. And in this cohort, it was significantly higher in the VB group than in the CS group (**Figure 3C**). This again indicated that differences in the mode of delivery possibly affects the bacterial species and thus the metabolite levels, and *Phocaeicola vulgatus* may be one of the key targets.

Bacteriophages family level were found to exhibit some correlations with delivery-associated SGBs (**Supplementary Figure 1H**). Myoviridae and Podoviridae showed significant positive correlations (*r* > 0.2, *P* < 0.05) with *Phocaeicola vulgatus*, while *Escherichia coli*_D displayed significant positive correlations (*r* > 0.2, *P* < 0.05) with Inoviridae and Myoviridae. Further suggesting an interactive relationship between these species and bacteriophages. We then performed similar correlation analyses of differential species versus bacteriophages in vOTUs levels (**Supplementary Table 13**). The network diagram showed that the VB group is equal to the complexity of the CS group (**Figure 5B**). In the VB group, 6 differential species were found to be significantly associated with bacteriophages (|*r*| > 0.2, *P* < 0.05). Among them, *Phocaeicola vulgatus*, *Escherichia coli_*D, and *Veillonella parvula*_A were significantly and positively associated with 7, 6, and 6 vOTUs, respectively. In the CS group, 5 species were found to be associated with different bacteriophages (|*r*| > 0.2, *P* < 0.05). *Phocaeicola vulgatus* and *Escherichia coli*_D were significantly positively correlated with 14, 13 vOTUs, respectively. As could be seen, *Phocaeicola vulgatus*, *Escherichia coli*_D were linked to delivery-related bacteriophages and were more sensitive in the CS group.

Combined with the SGBs-metabolites, SGBs-bacteriophage correlation analysis, we hypothesize that the mode of delivery affects the bacterial species in the infant’s gut, and that these species may further influence the metabolic profiles and bacteriophage structure and levels. Additionally, there may be some key species that have a wider range of influences.

## Discussion

The early development of the neonatal gut plays a crucial role in overall health and may even influence the susceptibility to atopic and allergic conditions later in life. Many current studies have emphasized the analysis of bacteria affecting the infant gut while neglecting the virome. Here, we collected fecal samples from mothers/infants through three regions with the aim of characterizing the microbiome in meconium and exploring which factors have an impact on the infant gut microbiota and bacteriophages in early development. Our results suggest a strong association between certain specific species and metabolite/phage vOTUs in the neonatal gut, influenced by mode of delivery. In addition, the health status of the mother may be critical for the early development of infant gut bacteriophages.

Our previous research has shown significant differences in the composition of gut microbiota between urban and pastoral populations in Inner Mongolia (Zhang et al., 2013). Additionally, other studies have indicated that geographic factors can influence the structure and composition of gut microbiota (Lu et al., 2021). Gut microbiota diversity is commonly considered as a key indicator of a healthy physiological state. In our cohort, we observed that geographic location exerted an impact on the diversity and structure of maternal gut microbiota and bacteriophage, but not the infant. However, neither geographic location, several maternal factors including mode of delivery, feeding practices, and maternal gestational diabetes also did not significantly affect the overall diversity of the infant’s gut microbiota. The limited impact of external factors on the overall diversity of neonatal meconium may be attributed to its initial low level of microbiota, making it challenging to detect significant variations. As the infants age, the gut microecology undergoes rapid colonization and growth, leading to the establishment of a stable and diverse gut microbiota. Unlike adults who have been acclimatized to their growing environment for many years, neonatal gut microbial communities are just beginning to be constructed, and geographic locations contain influences that are not yet sufficiently large to have a significant impact. The bacteria and bacteriophages they contain only need to fulfill the growth conditions of the current stage (e.g., catabolizing breast milk oligosaccharides) and do not require complex structures. However, differences in the structure of their gut microbes may only become apparent as they age and adapt for longer periods of time in their growing environment. This needs to be analyzed again on a time scale with longitudinal sampling.

The mode of delivery is widely acknowledged as a crucial factor influencing the initial acquisition and development of gut microbes, especially during the first year of life. Research findings have demonstrated that infants born through vaginal delivery are exposed to a diverse and abundant microbial community from the mother’s birth canal, leading to the establishment of a complex and varied gut microbiota. On the other hand, infants delivered via cesarean section might miss out on this vertical transmission pathway of microbes, resulting in a comparatively limited development of their gut microbial composition. *Bacteroidaceae*, including *Bacteroides* and *Phocaeicola*, are among the earliest microorganisms to colonize the gut of human infants. These microbial populations have the ability to be transmitted from mother to child during the early stages of development. It is well known that the mode of delivery effect in infants will be the largest the closer samples are taken to delivery. Within our cohort, we observed the presence of several *Bacteroides* and *Phocaeicola* members in the meconium of VB infants, which were significantly higher compared to CS infants. In a similar fashion, a recent study revealed the presence of *P. vulgatus* in both mothers’ skin within 10 minutes of birth and in meconium and stool samples of vaginally delivered infants at 3 months of age, and that *P. vulgatus* was highly prevalent in the meconium of both mothers and VB infants (Nilsen et al., 2023). Notably, *P. vulgatus* exhibited a remarkably high presence in almost all mothers included in this study (mean relative abundance 5.78%, prevalence 91.18%). We hypothesized that the mode of delivery might have influenced the vertical transmission of *P. vulgatus* from the mother’s intestine to the newborn. Whereas *P. vulgatus* is thought to degrade many plant-derived heteropolysaccharides, this process is important for later stages of infancy when solid foods are introduced. *B. thetaiotaomicron* is one of the most common bacteria found in the human gut microbiota, and it has also been found to have a number of genes in its genome that are specialized for polysaccharide digestion (Ryan et al., 2020). *B. xylanisolvens* is one of the strains of bacteria in the human gut that are involved in the breakdown of xylan (Chassard et al., 2008). And *Clostridium innocuum* (*C. innocuum*) has been identified as part of the normal gut microbiota (Crum-Cianflone, 2009). Nevertheless, the prevalence of *B. thetaiotaomicron*, *C. innocuum*, and *B. xylanisolvens* was relatively low (< 10%) and was observed only in VB infants. We attribute the variations in the occurrence of these species to individual variability.

In addition, *E. coli* levels were significantly higher in infants born by normal delivery than by cesarean section. Although *E. coli* is frequently pathogenic, it is also a crucial member of the human colonic microbial community, contributing to colonic fermentation and serving as an indicator of the fecal microbiota (Foster-Nyarko and Pallen, 2022). *E. coli*_D, observed in this study, could only be identified at the species level and it was not possible to determine whether it was a toxin-producing pathogenic *E. coli* strain. Since as early as 1976, studies have indicated that infants can acquire *E. coli* from the maternal vagina, with factors such as the duration of labor and infant swallowing being associated with this transmission. Research on infants born via normal delivery has demonstrated that about two-thirds of them typically acquire fecal *E. coli* from their mothers, while the remaining infants seem to acquire it from the surrounding environment (Bettelheim and Lennox-King, 1976). In this study’s cohort, *E. coli*_D was abundantly present in the gut of almost all mothers (mean relative abundance 4.63%, prevalence 100%) and infants (mean relative abundance 22.94%, prevalence 61.76%) (**Figure 1E**). The high occurrence of this species may be attributed to contamination in the hospital setting; however, it cannot be ruled out that it is due to a maternal-infant gut normal vertical transmission of microbiota. Maternity wards host a diverse array of *E. coli* serotypes and conducting a comprehensive assessment would involve collecting samples from the maternity ward environment and conducting meticulous biochemical and serologic studies. Similar to the case of *P. vulgatus*, *E. coli*_D had a higher mean relative abundance in mothers. The distribution of these two species may suggest that VB infants may have a greater chance of inheriting a dominant species in the mother’s gut.

In our work, certain members of the *Veillonella* genus (*V. parvula*_A, *V. infantium*, and *V. dispar*) were found to be significantly more abundant in infants delivered via cesarean section compared to those born through normal births. *Veillonella* is commonly present in the oral microbiota (Zhou et al., 2021) and has been associated with bacteremia and infections in some cases (Cobo et al., 2020). However, it is not a coincidence that they have been detected in the intestines of infants. In their study utilizing 16S rRNA sequencing, Liu et al. identified *Veillonella* as the 7th most abundant genus in the feces of 6-week-old infants. Interestingly, they observed a higher abundance of *Veillonella* in the intestines of infants born via cesarean section compared to those born through normal delivery. In a global meta-analysis, *Veillonella* was also found to be enriched in the feces of infants born by cesarean section (Wang et al., 2021). Additionally, a significant difference in the relative abundance of *Veillonella* was noted between exclusively breastfed and MF infants, indicating that the presence of *Veillonella* might be influenced by both the mode of delivery and the type of feeding (Liu et al., 2019). In addition, *Clostridium_*P *perfringens* (*C. perfringens*) and *Staphylococcus hominis* (*S. hominis*) were also more enriched in cesarean section infants. According to an authoritative review, infants born through vaginal delivery typically harbor more maternal vaginal and gut-derived microorganisms, such as *Bacteroides*, *Bifidobacterium*, and *Escherichia*. On the other hand, infants delivered via cesarean section tend to have a microbiota that reflects maternal skin surfaces (*Staphylococcus*, *Corynebacterium*), oral cavity (*Veillonella*), and potentially pathogenic bacteria associated with hospital environments (*Klebsiella*, *Clostridia*) (Xiao and Zhao, 2023).

In addition, we found that *K. pneumoniae* was significantly higher in mixed-fed infants, whereas *Corynebacterium kefirresidentii*, *Staphylococcus lugdunensis*, and *Streptococcus infantis*_I appeared only in the feces of exclusively feces of breastfed infants. *K. pneumoniae*, a prevalent bacterium in nosocomial infections, has been linked to sepsis and necrotizing small bowel colitis in premature infants (Paveglio et al., 2020; El Manouni El Hassani et al., 2021). In a study conducted by Li et al (Li et al., 2020), they observed elevated levels of *Klebsiella* in the stool samples of MF infants. This analysis was based on 16S rRNA gene sequencing of fecal samples collected from infants ranging from 16 days to 295 days of age. It is noteworthy that *K. pneumoniae*, *Staphylococcus lugdunensis*, and *Rothia mucilaginosa* are influenced not only by the mode of breastfeeding, but also by geographic location. Research has indicated a notable rise in opportunistic pathogens within contemporary infant microbial communities. These pathogens primarily consist of *Enterococcus faecalis*, *Klebsiella michigans*, *Klebsiella pneumoniae*, and *Staphylococcus epidermidis*. However, it has been observed that these opportunistic pathogens tend to decline and eventually disappear after the first year of life (Backhed et al., 2015; Lugli et al., 2023). Chu et al. (Chu et al., 2017) analyzed 117 meconium samples and further identified *Escherichia*, *Klebsiella*, and *Bacteroides* with high specificity in newborns. These findings may shed light on why *K. pneumoniae* and *Staphylococcus lugdunensis* were exclusively detected in the feces of infants from certain cities or only found in infants who were exclusively breastfed, as observed in our study.

Our findings align with these existing perspectives, further supported that vaginal delivery and breastfeeding are beneficial to the early development of infants. But it is essential to acknowledge that the small sample size and data collected from different hospitals may introduce some limitations and potential errors in the results. However, it is undeniable that there are differences in the microbiota of newborns born via cesarean section and vaginal delivery, even if these changes are limited to a few taxa and may be transient. Furthermore, there have been proposals that the effects of mode of delivery are mostly resolved during infancy, and any potential long-term impacts on metabolism and immune system development in later life have not been definitively linked (Sassin et al., 2022). As a result, future research on the microbial structure of the infant gut would benefit from larger and longer-term cohort studies to gather more robust scientific evidence and further understand the role of gut microbiota in promoting healthy infant development.

The gut microbiota plays a crucial role in producing various metabolites and small molecules, which act as signaling molecules and substrates for host metabolic reactions (Krautkramer et al., 2021). A Meta-analysis showed that infant purine nucleotides, proteinogenic amino acids and folate biosynthesis were particularly enriched compared to, maternal metabolic pathways (Wang et al., 2021). We predicted bioactive metabolites in the infant gut. Among the differential metabolites, the metabolism observed in VB infants seems to be associated with creatinine, purine nucleotides, lipids, and other related pathways. On the other hand, CS infants may have a higher prevalence of metabolic pathways related to pyrimidine nucleotides, amino acids, vitamins, and other compounds. In our further correlation analysis, we observed strong positive correlations between *P. vulgatus* and certain metabolites, such as hydrocinnamic.acid, X3.methyladipate.pimelate, and inosine, which were found to be significantly enriched in VB infants. On the other hand, *P. vulgatus* showed strong negative correlations with metabolites like taurine and erythronic acid, which were significantly enriched in the CS group. These findings suggest that *P. vulgatus* may play a crucial role as a keystone species in the infant gut, influencing multiple metabolic pathways. *B. xylanisolvens*, *B. thetaiotaomicron* and phenylacetate all showed a positive correlation in VB infants, while *Bacteroides* spp. may be the main microbial source of phenylacetic acid production in humans (Russell et al., 2013). Likewise, this consistent relationship between the levels of differential metabolites and specific bacterial groups was also observed in the CS group. These findings suggest that the mode of delivery may impact the functionality of the core microbiota in infants, leading to changes in the levels of associated metabolites. However, the data we detected can only represent the metabolite profiles within a short period of time after delivery. Infant gut metabolic profiles change at different stages, and the metabolic profiles are gradually adjusted and shifted as the infants grow and develop and as their dietary habits change.

Currently there is little known about how and when the neonatal gut bacterial microbiota and virome are acquired. Sequencing of the virome in the intestine of infants at 1 to 2 weeks of age revealed that the diversity of the infant virome is notably low, with the majority of recognizable sequences resembling bacteriophages. These bacteriophages have the potential to impact the diversity and structure of the microbial community in the developing infant’s gut (Breitbart et al., 2008). Bacteriophages and bacteria are in a predator-prey relationship, whereby one phage will only exclusively parasitize or infect one type or similar bacterial host (Kirsch et al., 2021). Bacteriophage-bacteria interactions in the gut lead to different ecological dynamics, and currently hypothesized kinetic relationships include Red Queen dynamics, Kill-the-winner dynamics, and Piggyback-the-winner dynamics. A 2015 study concluded that the population dynamics of phage-bacteria interactions in infant gut follow Lotka Volterra’s Kill-the-winner dynamics model, meaning that phage-bacteria relationships have a high predator-low prey dynamic from birth (Lim et al., 2015). Liang et al (Liang et al., 2020) also found a strong positive correlation between the abundance of gut bacteria in neonates and the abundance of bacteriophages in this virus. Similarly, in our cohort, the gut bacterial diversity and composition of neonates was strongly correlated with their bacteriophage diversity and composition, which indirectly indicated that bacteriophages are highly infectious for their host bacteria. The dynamics of association between bacteriophages and host bacteria from individuals from birth to adulthood need to be further explored. Our results are contributing to the construction of a system of knowledge on the initial ecological relationships of infant bacteriophages-bacteria.

Adult bacteriophage diversity (species) and structure are altered in certain disease states, such as ulcerative colitis and Crohn’s disease, etc (Mirzaei and Maurice, 2017). Studies on the association of gut phages with type 2 diabetes (T2D) have shown an increase in the number of gut phages in patients with T2D compared to healthy individuals (Ma et al., 2018). T2D and the complications of nephropathy or obesity are characterized by a significant reduction in the diversity of gut viruses, a change in the specific viral species, and a disruption of virus-bacterial correlation (Yang et al., 2021; Fan et al., 2023). However, there is a paucity of clarity regarding the external factors that influence the development of infant phage structure. Phage colonization of the infant gut is stepwise, with temperate phage replication in human cells induced primarily by pioneer bacteria (0-1 month); the second phase (1-4 months) is regulated by breastfeeding, with different structures induced by different feeding patterns (Liang et al., 2020). In addition, the diversity and structure of enteroviruses were found to be different among infants with different modes of delivery at 1 year of age (McCann et al., 2018). Identical twins have more similar virome than unrelated individuals, suggesting that genetic factors play a role in bacteriophages composition (Lim et al., 2015). In another study, infants’ lack of a core temperate virome of a specific viral family may be a risk factor for the development of asthma in preschoolers (rather than bacteria), and this risk may be influenced by host genetic factors (Leal Rodriguez et al., 2024). Our results also supported the effect of breastfeeding delivery mode and feeding mode on bacteriophage levels, although their long-term regulation of infant bacteriophages could not be discerned due to the short sampling period. We also found that gestational diabetes reduced maternal bacteriophages diversity as well as reported for the first time that maternal gestational diabetes may affect initial infant bacteriophages diversity. We hypothesize that gestational diabetes may affect the maternal gut bacteriophages, which in turn may have a genetic impact on the newborn’s gut bacteriophages. On the other hand, gestational diabetes and other maternal disease states may have an impact on host gut microbial interactions. Although our sample size was unbalanced in terms of subgroups (10 pairs in the GD group and 24 pairs in the none-GD group), it adds to the void regarding the effect of maternal health on the infant’s gut virome. In the future, research on maternal and neonatal health should focus more on the analysis of the virome.

As we mentioned, our study has some limitations. First, multicenter sampling could not ensure the consistency of each doctor’s actions. Additionally, there could be other potential factors not explored in our study, such as the delivery environment, maternal antibiotic use, and various maternal health conditions (e.g., hypertension, anemia, hypothyroidism), which might have influenced the results to some extent. Moreover, the small sample size of our study and the imbalance in sample sizes when grouping according to different factors could introduce bias in the results. These limitations highlight the need for larger and more comprehensive studies to obtain a more robust understanding of the complex interactions between the gut microbiome and various. Most importantly, due to the limitations of sequencing depth we did not find gut bacterial or bacteriophage transmission phenomena to further demonstrate the genetic relationship between mother and infant. We noticed that the results of bacteriophages correlation analysis showed different significance when using different levels of classification units. This indicated that the assembly technology and the completeness of the database would greatly affect the accuracy of the analyzed results, and our current technology for mining the bacteriophages group is still only the tip of the iceberg. In the future, more comprehensive and accurate results may be obtained through the improvement of sequencing technology and analyzing technology.

## Conclusion

In conclusion, our study highlighted the important influence of maternal factors (mode of delivery, mode of feeding, gestational diabetes mellitus) on the microbial structure of the neonatal gut. Certain key species (e.g., *Phocaeicola vulgatus*) are affected by the mode of delivery, interact with specific bacteriophages, or have different metabolic effects. These changes may have implications for the development and functional integrity of the gut in later infancy. This study provided new evidence for the study of the infant microbiome, fills a void in the analysis of the infant gut virome, and emphasizes the importance of maternal health on the initial infant gut virome.

## Data Availability

The data presented in the study are deposited in the NCBI (National Center of Biotechnology Information) repository, accession number PRJNA1065277, https://www.ncbi.nlm.nih.gov/.

## Supplementary material

Supplementary Figure 1
**(A)** Family-level taxonomic distribution of the phageome metagenome. **(B)** Shannon diversity index and **(C)** PCoA analysis of mother/infant gut bacteriophage families in various cities. **(D)** Bacteriophages vOTUs Shannon index of mothers in GD and no GD groups. **(E)** Anosim analysis demonstrated within- and between-group differences in infant gut microbes by different factors. *R* < 0 indicates that the within-group difference is greater than the between-group difference, and *R* > 0 indicates that the within-group difference is less than the between-group difference. **(F, G)** The significant differential bacteriophage vOTUs in the infant gut related to mode of feeding and whether the mother is GD. **(H)** Significant correlations between differential SGBs and bacteriophage families associated with delivery. **(I)** Effect of gestational diabetes mellitus on the bacteria Shannon index- bacteriophages Shannon index correlation. **(J)** Correlation analysis of mother-infant bacteriophages diversity in the GD and none-GD groups. Es, Enshi; Hlb, Hulunbuir; Hh, Hohhot. RPKM, Reads Per Kilobase per Million mapped reads. CS, Caesarean section; VB, Vaginal birth; BF, Breast feeding; MF, Mixed feeding; GD, Gestational diabetes; none-GD, None-Gestational diabetes. Wilcoxon test was used to evaluate statistical differences; *P* < 0.05 was considered statistically significant [**P* < 0.05; ***P* < 0.01].
