# Supplementary figures and images for: Delivery mode and maternal gestational diabetes are important factors in shaping the neonatal initial gut microbiota
Source: Front Cell Infect Microbiol. 2024 Aug 29;14:1397675. doi: 10.3389/fcimb.2024.1397675 (PMC11390658; doi:10.3389/fcimb.2024.1397675)

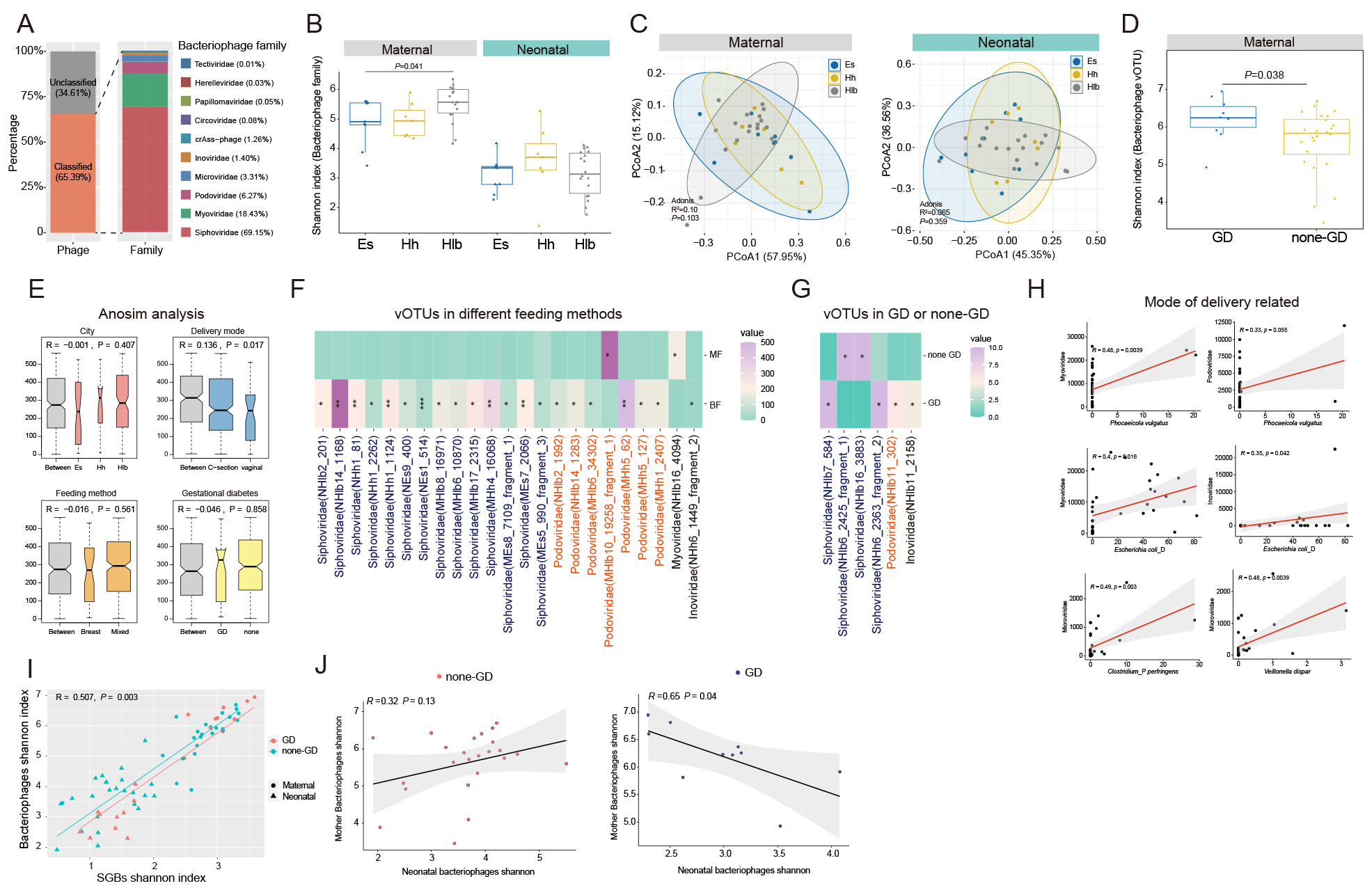

Supplement: Supplementary Figure 1 — (A) Family-level taxonomic distribution of the phageome metagenome. (B) Shannon diversity index and (C) PCoA analysis of mother/infant gut bacteriophage families in various cities. (D) Bacteriophages vOTUs Shannon index of mothers in GD and no GD groups. (E) Anosim analysis demonstrated within- and between-group differences in infant gut microbes by different factors. R < 0 indicates that the within-group difference is greater than the between-group difference, and R > 0 indicates that the within-group difference is less than the between-group difference. (F, G) The significant differential bacteriophage vOTUs in the infant gut related to mode of feeding and whether the mother is GD. (H) Significant correlations between differential SGBs and bacteriophage families associated with delivery. (I) Effect of gestational diabetes mellitus on the bacteria Shannon index- bacteriophages Shannon index correlation. (J) Correlation analysis of mother-infant bacteriophages diversity in the GD and none-GD groups. Es, Enshi; Hlb, Hulunbuir; Hh, Hohhot. RPKM, Reads Per Kilobase per Million mapped reads. CS, Caesarean section; VB, Vaginal birth; BF, Breast feeding; MF, Mixed feeding; GD, Gestational diabetes; none-GD, None-Gestational diabetes. Wilcoxon test was used to evaluate statistical differences; P < 0.05 was considered statistically significant [*P < 0.05; **P < 0.01]. [file Image1.tif]
